# InDel marker based genetic differentiation and genetic diversity in traditional rice (*Oryza sativa* L.) landraces of Chhattisgarh, India

**DOI:** 10.1371/journal.pone.0188864

**Published:** 2017-11-30

**Authors:** Parmeshwar Kumar Sahu, Suvendu Mondal, Deepak Sharma, Gautam Vishwakarma, Vikash Kumar, Bikram Kishore Das

**Affiliations:** 1 Department of Genetics and Plant Breeding, Indira Gandhi Krishi Vishwavidyalaya, Raipur, Chhattisgarh, India; 2 Nuclear Agriculture and Biotechnology Division, Bhabha Atomic Research Centre, Trombay, Mumbai, India; National Cheng Kung University, TAIWAN

## Abstract

Rice has been cultivating and utilizing by humans for thousands of years under diverse environmental conditions. Therefore, tremendous genetic differentiation and diversity has occurred at various agro-ecosystems. The significant *indica–japonica* differentiation in rice provides great opportunities for its genetic improvement. In the present investigation, a total of 42 polymorphic InDel markers were used for differentiating 188 rice landraces and two local varieties of Chhattisgarh, India into *indica* and *japonica* related genotypes based on ‘InDel molecular index’. Frequency of *japonica* alleles varied from 0.11 to 0.89 among landraces. Results revealed that 104 rice landraces have *indica* type genetic architecture along with three tested *indica* cultivars Swarna, Mahamaya and Rajeshwari. Another 60 landraces were placed under ‘close to *indica*’ type. It was found that three rice landraces *i*.*e*. Kalajeera, Kapri, Tulsimala were ‘close to *japonica*’ type and 21 landraces were ‘intermediate’ type. The result from the calculation of ‘InDel molecular index’ was further verified with STRUCTURE, AMOVA, PCA and cluster analysis. Population structure analysis revealed two genetically distinct populations within the 190 rice landraces/genotypes. Based on AMOVA, ‘intermediate’ type, ‘close to *japonica*’ type and Dongjinbyeo (a *japonica* cultivar from Republic of Korea) displayed significant genetic differentiation (ɸ_PT_ = 0.642, P = 0.000) from ‘*indica*’ and ‘close to *indica*’ groups. The PCA scatter plot and dendrogram demonstrated a clear pattern of two major group differentiations. ‘Close to *japonica*’ type and ‘intermediate’ type landraces/genotypes were grouped with Dongjinbyeo and formed a separate cluster at 30% Jaccard’s similarity level from rest of the landraces/genotypes which were ‘close to *indica*’ or ‘*indica*’ type. Such a significant genetic differentiation among the locally adapted landraces could be exploited for the development of rice varieties introgressing higher yield potential and better plant types of *japonica* type as per the need of consumers and rice traders.

## 1. Introduction

Rice (*Oryza sativa* L.) is one of the most important food crops in the world and supports the requirement of staple food for almost half of the world’s population. It is cultivated in about 163.2 million ha of land and produced 751.9 million tons (in form of paddy) worldwide [1]. Of the 25 species in rice, only two species, *Oryza sativa* and *Oryza glaberrima* are cultivated [2,3]. The *Oryza sativa* is the most commonly grown species throughout the globe. While, *Oryza glaberrima* is grown mainly in African sub-continent. *Oryza sativa* is differentiated into three sub-species based on geographical and agro-climatic requirements *viz*., *indica*, *japonica* and *javanica*. Significant morphological, physiological and biochemical differences between *indica* and *japonica* subspecies makes them tremendously unique. The genetic divergence among the subspecies of *Oryza sativa* L. is valuable for further genetic improvement in cultivated rice. Xiong et al. [4] suggested that the recombinogenic segregating progenies derived from *indica*–*japonica* hybridization can provide valuable genetic materials for rice breeders in selecting traits for meeting breeding goals. Further, the inter-subspecies hybridization between *indica* and *japonica* rice has enough potential to generate extremely strong hybrid vigour (heterosis) which attracted the rice breeders for developing ‘super hybrid rice’ [4]. However, the problem of partial sterility in the inter-subspecies crosses limits their effective utilization. The genetic differentiation between *indica* and *japonica* rice provides information about adaptive evolution in plant species under the changing environments which may in turn help rice breeders for resolving partial sterility in inter-subspecies hybridization in rice [5,6].

It is essential to accurately identify the *indica* and *japonica* rice genotypes used for genetic improvement of rice and to understand the mechanism of *indica-japonica* genetic differentiation during domestication process. Generally, *indica* and *japonica* rice varieties are identified by variation in morphological traits (plant height, plant type, status of pubescence of plants, and type of grains) in combination with some physiological and biochemical features (winter hardiness, starch types and phenol response in grains). Of these various attributes, six parameters were used by Cheng et al. [7] for differentiation of these two rice types. These six parameters which are used for *indica/ japonica* differentiation is popularly known as ‘Cheng’s Index’. However, this method is laborious and largely affected by environmental conditions. Therefore, use of molecular markers associated with this differentiation might help to correctly assign rice genotypes into these two groups. ‘InDel molecular index’ which was proposed by Lu et al. [8] is used in recent days for the identification of *indica* or *japonica* rice varieties. It is based on the proportion of *indica* or *japonica* allele (band) amplified from the rice genotypes using InDel markers. Polymorphism of InDel markers is based on insertion and deletion (InDel) fragments obtained in the comparison of genomic DNA sequences between the typical *indica* rice genotype 93–11 and *japonica* rice genotype Nipponbare [9]. Apart from the identification of *indica* and *japonica* cultivars, InDel markers could promote studies of genetic diversity and differentiation of rice in depth, and help to construct a genome-wide rice DNA polymorphism database [10]. These InDel markers were used to distinguish basmati from other aromatic rice cultivars [11] and to differentiate wild *Oryza* species [12]. Xiong et al. [4] and Yamaki et al. [13] successfully utilized InDel markers for discriminating different sub-species of *Oryza sativa* L. as well some wild rice species.

India has a rich resource of rice germplasm. The available cultivars, genotypes, breeding lines, mutants and land races in India impart a greater genetic diversity in the cultivated rice germplasm. Moreover, state-wise differential choices for rice has made a selective evolution of rice landraces in a region/state specific manner. Das et al. [14], Chowdhary et al. [15] and Roy et al. [16] conducted genetic diversity and population structure analysis in rice germplasm of North-Eastern states of India that showed a great extent of genetic diversity among those rice landraces or genotypes. Chhattisgarh is traditionally known for a rich heritage of indigenous rice varieties that were adapted to different agro-ecosystems. It is traditionally known as the ‘Rice Bowl of India’ due to its richness in diversity for different agro-economic traits. Over 23,250 rice germplasm lines have been recorded in the region [17,18]. These are the result of centuries of rice farming by indigenous communities through selection and adaptation to a variety of soil, water and micro-ecosystems. Rice is the dominating crop of this state where small farmers and tribal communities grow most of the traditional indigenous rice varieties. Selective breeding, random mutation as well as frequent hybridization between the landraces and wild relatives over a long time ensured the accumulation of a high phenotypic as well as genetic diversity [19]. Retention of immense genetic diversity is not only significant in terms of evolutionary potential to withstand diverse selection regimes, but also has its important implications in rice breeding towards furnishing new stress resistance genes for crop improvement [20].

Rice genetic resources are the common heritage of mankind and essential for genetic improvement of rice and developing desired rice varieties. However, the genetic diversity in rice has been drastically reduced due to aggressive introduction of modern varieties/ high yielding varieties and disappearance of traditional/ indigenous landraces. Due to the narrow genetic base, modern rice varieties are less adaptable to varying agro-climatic conditions. Their yield performance, biotic and abiotic stress tolerance ability and other agronomic traits can be genetically broken down due to narrow genetic base. Therefore these cultivars used to disappear after few years of cultivation. Thus, exploration of genetic resources like landraces and indigenous rice varieties is an important and urgent issue in crop breeding for developing durable, climate resilient and high yielding rice varieties with desired quality attributes. Towards these understanding of genetic relationship among rice landraces will help their proper utilization in rice breeding program. InDel markers are developed based on either an insertion or deletion in genomic sequence and they can be used as genetic markers in natural populations, especially in phylogenetic studies [12,21]. Therefore, InDel marker based genetic differentiation and genetic diversity study will be useful for further genetic improvement of rice landraces.

In the present study, we have used 190 rice landraces/genotypes of Chhattisgarh, India to detect genetic relationships, population structure and genetic diversity among them. Further, these rice landraces were classified into *indica* and *japonica* related genotypes based on ‘InDel molecular index’. The study revealed immense potential to use these diverse landraces for genetic improvement of rice as well as for further enhancement of yielding potential.

## 2. Materials and methods

### 2.1. Experimental plant materials

**Fig 1:**
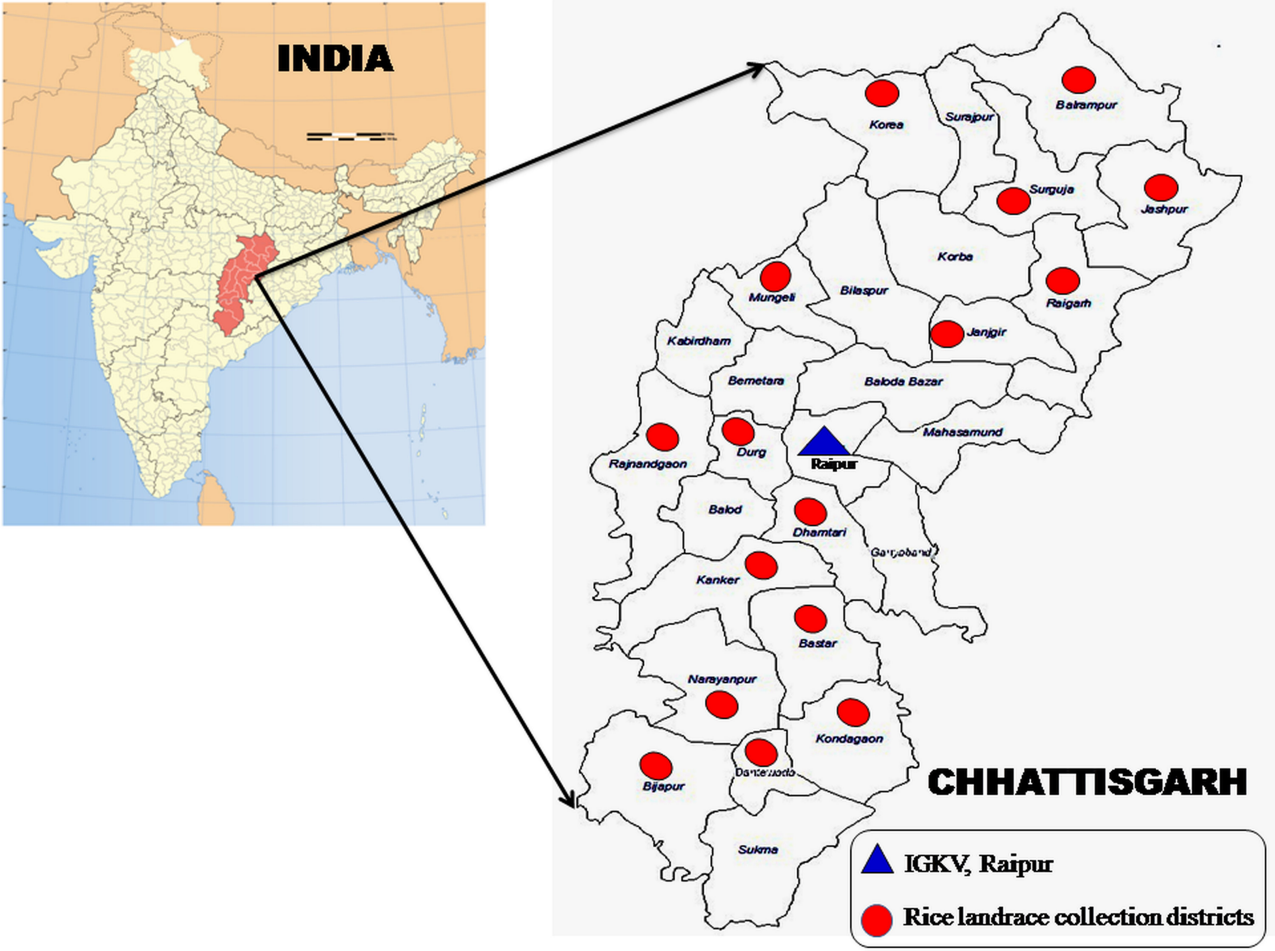
Pictorial map of collection sites of 190 rice landraces/genotypes from Chhattisgarh states in India. Note: The map was downloaded from https://upload.wikimedia.org/wikipedia/commons/7/79/India_Chhattisgarh_locator_map.svg and was reproduced here with due permission from the curator (Courtesy to arun.planemad@gmail.com).

Plant materials include 188 rice landraces (S1 Table) that were collected from different tribal districts of Chhattisgarh state in India (Fig 1). The study also includes a reference *japonica* variety ‘Dongjinbyeo’ from Republic of Korea, a reference *indica* variety ‘Swarna’ from India and two locally cultivated varieties Mahamaya and Rajeshwari. Seeds of rice cultivar Dongjinbyeo were procured from Advanced Radiation Technology Institute, Korea Atomic Energy Research Institute, Jeongeup, Republic of Korea. The seeds of Swarna, Mahamaya and Rajeshwari were taken from department of genetics and plant breeding, Indira Gandhi Krishi Vishwavidyalaya, Raipur, Chhattisgarh, India.

### 2.2. DNA extraction

Seeds of each rice genotype were germinated in a growth chamber with 10 hours light, 85% relative humidity and 30°C temperature. At the three-leaf stage, leaf tissues from five seedlings of each genotype were collected for DNA extraction. The total genomic DNA was then isolated from this leaf tissue using GenElute^TM^Plant Genomic DNA mini prep kit (Sigma, USA) following the manufacturer’s protocol.

### 2.3. InDel locus selection

A total of 45 InDel primer pairs distributed over 12 rice chromosome were used initially for detection of polymorphism between eight genotypes that included Dongjinbyeo and Swarna. These 45 InDel primer pairs were developed by Shen et al. [9] and successfully utilized and validated by Lu et al. [8] and Xingxing et al. [10] for differentiating the *indica* and *japonica* rice varieties. The InDel markers were developed based on differences in DNA sequences of the total genome between the typical *indica* rice genotype (93–11) and the typical *japonica* genotype (Nipponbare), and particularly represented variation between *indica* and *japonica* [8,9]. Among 45 InDel markers, 42 were found to be polymorphic between Dongjinbyeo and Swarna and also in rice landraces. Details about these InDel primer pairs are given in Table 1.

**Table 1:** Details of InDel markers used in the present investigation.

S. No.	Marker name	Chr No.	Allele Size (bp)	InDel size (bp)	PIC	Rp	NPA	PPA	EMR	MI
1	R1M7	1	195 and 157	38	0.05	0.11	2	1	2	0.1
2	R1M30	1	248 and 202	46	0.38	1.24	2	1	2	0.76
3	R1M37	1	167	-	0.01	0.01	2	1	2	0.02
4	R1M47	1	162 and 110	52	0.2	0.47	2	1	2	0.4
5	R2M10	2	188 and 141	47	0.09	0.11	2	1	2	0.18
6	R2M24	2	167 and 136	31	0.21	0.49	2	1	2	0.42
7	R2M26	2	180 and 146	34	0.24	0.64	2	1	2	0.48
8	R2M37	2	211 and 151	60	0.42	1.85	2	1	2	0.85
9	R2M50	2	249 and 210	39	0.21	0.48	2	1	2	0.42
10	R3M10	3	194 and 171	23	0.03	0.06	2	1	2	0.06
11	R3M23	3	226 and 189	37	0.21	0.47	2	1	2	0.42
12	R3M30	3	186 and 163	23	0.08	0.17	2	1	2	0.16
13	R3M37	3	282, 240 and 194	42	0.48	1.46	3	2	6	2.88
14	R3M53	3	209 and 175	34	0.44	1.46	2	1	2	0.89
15	R4M13	4	184 and 178	6	0.2	0.46	2	1	2	0.41
16	R4M17	4	227 and 175	52	0.44	1.85	2	1	2	0.89
17	R4M30	4	165 and 130	35	0.49	1.78	2	1	2	0.98
18	R4M43	4	208 and 171	37	0.23	0.54	2	1	2	0.47
19	R4M50	4	173 and 144	29	0.37	0.99	2	1	2	0.74
20	R5M13	5	213 and 178	35	0.23	0.52	2	1	2	0.45
21	R5M30	5	222 and 179	43	0.46	1.45	2	1	2	0.91
22	R6M14	6	256 and 221	35	0.43	1.29	2	1	2	0.85
23	R6M44	6	164 and 128	36	0.2	0.47	2	1	2	0.4
24	R7M7	7	213 and 145	68	0.36	0.96	2	1	2	0.73
25	R7M20	7	225 and 212	13	0.98	0.02	2	1	2	1.96
26	R7M37	7	182 and 164	18	0.03	0.07	2	1	2	0.06
27	R8M23	8	168 and 127	41	0.01	0.02	2	1	2	0.02
28	R8M33	8	213 and 175	38	0.44	1.39	2	1	2	0.88
29	R9M10	9	184 and 143	41	0.39	1.04	2	1	2	0.78
30	R9M20	9	187 and 137	50	0.22	0.48	2	1	2	0.44
31	R9M30	9	197 and 165	32	0.27	0.65	2	1	2	0.54
32	R9M42	9	231 and 218	13	0.03	0.06	2	1	2	0.06
33	R10M10	10	177 and 138	39	0.43	1.86	2	1	2	0.86
34	R10M17	10	158 and 128	30	0.07	0.15	2	1	2	0.14
35	R10M30	10	206 and 189	17	0.47	1.85	2	1	2	0.93
36	R10M40	10	164 and 134	30	0.01	0.02	2	1	2	0.02
37	R11M23	11	253 and 215	38	0.19	0.44	2	1	2	0.39
38	R11M40	11	185 and 141	44	0.38	1.18	2	1	2	0.76
39	R12M10	12	268 and 221	47	0.01	0.05	2	1	2	0.02
40	R12M27	12	185 and 156	29	0.35	0.9	2	1	2	0.7
41	R12M33	12	264 and 219	45	0.5	1.8	2	1	2	1
42	R12M43	12	204 and 174	30	0.2	0.45	2	1	2	0.4

Note: PIC = Polymorphic information content, Rp = Resolving power, NPA = Number of polymorphic amplicon, PPA = Proportion of polymorphic amplicon, EMR = Effective multiplex ratio, MI = marker index.

### 2.4. PCR amplification and electrophoresis analysis

PCR reactions were performed in 10 μl mixture containing 4 μl (2.5 ɳg/ μl) template DNA, 2 μl of 5x assay buffer, 2 mM MgCl_2_ (Promega, Madison, USA), 0.2 μM of each forward and reverse InDel primer, 200 μM dNTPs (Roche, Indianapolis, USA) and 1.5 U of *Taq* DNA polymerase (BRIT, Mumbai, India). PCR reactions were carried out in a thermal-cycler (Eppendorf, Hamburg, Germany). The amplification profile consisted of initial denaturation for 5 min at 95°C; 10 cycles of 30s denaturation at 95°C, 30s annealing at 55°C followed by decrement of temperature @ -0.5°C per cycle (for 10 cycle) and 40s extension at 72°C. Remaining 25 cycles were used to amplify DNA with denaturation at 94°C for 30s, 30s annealing at 50°C and amplification for 40s at 72°C. After that, final extension was carried out at 72°C for 5 min. Initially, polymorphism and allele size of all the InDel primer pairs were checked on capillary electrophoresis (Qiagen Pvt. Ltd., Hamburg, Germany) by using a reference *indica* variety Swarna and a reference *japonica* variety Dongjinbyeo along with six highly diverse landraces identified by SSR based molecular diversity in a previous laboratory experiment (unpublished). Forty two InDel primer pairs showing polymorphism between the Swarna and Dongjinbyeo and other six landraces were used for genotyping of the whole set of landraces. PCR products were resolved on 2.5% agarose gel (containing 1.75% Methaphor® agarose + 0.75% normal agarose) (Lonza, NJ, USA). After separation, gel image was taken with a gel documentation unit (Syngene, Cambridge, UK). The null alleles and heterozygous alleles were confirmed after several repetitions with different amplification conditions to ensure that no reaction failure existed.

### 2.5. Calculation of *indica* specific and *japonica* specific allelic frequency

**Fig 2:**
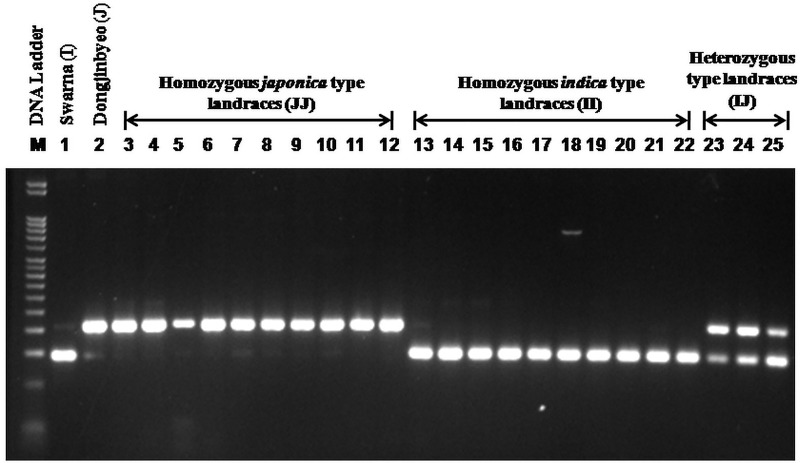
Amplification profile of the InDel marker, R2M37 in Dongjinbyeo, Swarna and landraces of Chhattisgarh, India. Note: M = 50–2000 bp DNA Ladder (Step ladder 50 bp, Sigma, USA), 1 = Swarna, 2 = Dongjinbyeo, 3 = Anjani, 4 = Bathrash, 5 = Jonyaphool, 6 = Laxmibhog, 7 = Tulsimongra, 8 = Dhaura Mundariya, 9 = Jhimipras, 10 = Pangudi Goindi, 11 = Agyashal, 12 = Jauphool, 13 = Pratiksha, 14 = Bhadvel, 15 = Bhajna, 16 = Sawani, 17 = Safri, 18 = Dubraj, 19 = Kalajeera, 20 = Dhaura Mundariya 2, 21 = Gangachur, 22 = Karhani, 23 = Ratajhinga, 24 = Alsenga, 25 = Gurkamal Dhan.

The InDel markers were co-dominant, therefore, the electrophoretic banding patterns were scored as either the homozygous *indica*-genotype (II), homozygous *japonica*-genotype (JJ), or heterozygous *indica*–*japonica*-genotype (IJ). The electrophoretic banding patterns of Swarna and Dongjinbyeo were used as a reference for determining the *indica* or *japonica* genotype, respectively, at a particular InDel locus. At any given InDel locus, if examined rice landrace showed banding pattern similar to that of Swarna, it was scored as homozygous *indica* genotype (II). If the examined rice landrace showed banding pattern similar to that of Dongjinbyeo, it was scored as homozygous *japonica* genotype (JJ) at the given InDel locus. If the examined sample showed both bands that were identical to Swarna and Dongjinbyeo at a particular InDel locus, this locus was determined as heterozygous *indica*–*japonica* genotype (IJ) (Fig 2).

To analyze genetic relationships of all rice landraces, an allele size data matrix of genotype banding patterns obtained from all rice landraces was converted to a binary (0, 1) data matrix. The identification of *indica* or *japonica* characteristics and the evolutionary relationship of each rice landrace were estimated based on the average allelic frequencies of *indica* specific alleles (F_i_) or *japonica* specific alleles (F_j_) across 42 InDel loci according to the method developed by Lu et al. [8]. The calculation for the *indica* or *japonica* allelic frequency (F_i_ or F_j_) of a particular rice landrace at all examined InDel loci was done by following formula of Lu et al. [8]. Frequency of *indica* alleles (F_i_) was calculated as: (2∑X_ii_ + ∑X_ij_)/2N. Similarly frequency of *japonica* alleles (F_j_) was calculated as: (2∑X_jj_ + ∑X_ij_)/2N. Where X_ii_ indicates the homozygous *indica* genotype (II), X_jj_ indicates the homozygous *japonica* genotype (JJ), X_ij_ indicates the heterozygous *indica*-*japonica* genotype (IJ) at a given InDel locus of a particular rice sample scored based on the electrophoresis banding pattern, respectively; N indicates the total number of InDel loci examined.

### 2.6. STRUCTURE, cluster analysis and principle component analysis

Population structure was examined using Bayesian model-based approach in STRUCTURE V2.3.4 [22,23]. The number of clusters tested here ranged from 1 to 10. The analysis was performed using 5 replicate runs per K value; a burn-in period of 100000, MCMC replications after burn-in was 100000. A model permitting for admixture and correlated allele frequency was used for the analysis. Structure harvester program was used to determine the final K value based on LnP(D) and Evano’s ΔK [24].

The binary data matrix of all InDel markers in all the rice landraces including Swarna and Dongjinbyeo was subjected to cluster analysis by following UPGMA (unweighted pair group method with arithmetic mean) method based on Jaccard’s similarity coefficient as genetic distance measurement with the help of PAST 3.15 [25]. A principle component analysis for assessing genetic diversity among landraces was also conducted based on variance-covariance matrix using PAST 3.15 software [25]. The principle component axes were drawn according to eigenvalues. By using the latitude, longitude and altitude information of collection sites of landraces a principal coordinate analysis (PCoA) analysis for geographical diversity was also conducted by PAST 3.15.

### 2.7. Marker utility information

In order to obtain measures of utility of the marker system, the polymorphic information content (PIC), Effective Multiplex Ratio (EMR), Marker Index (MI) and Resolving Power (Rp) were calculated for each marker according to Powell et al. [26]. For each locus PIC was calculated as 1-∑*pi*^2^ (where *pi* is the frequency of i^th^ allele). EMR was calculated as the product of the fraction of polymorphic loci and the number of polymorphic loci for an individual assay [27]. MI was calculated as the multiplication of product of PIC and EMR. Resolving power was calculated as R_p_ = ∑I_b_ [28], where I_b_ is the band informativeness. I_b_ was calculated as I_b_ = 1-(2* │0.5-p│), where p is the proportion of the genotype containing the band. When an allele was found in less than 5% of the landraces under study, it was considered as rare allele [29].

### 2.8. Analysis of molecular variance (AMOVA)

Based on the ‘InDel molecular index’ the whole collection of rice landraces/genotypes was divided into two groups or populations. The landraces which were ‘close to *japonica*’ type and ‘intermediate’ type were brought under population-2. The remaining landrace which were ‘close to *indica*’ and ‘*indica*’ type were grouped in population-1. Genetic differentiation between these two groups/populations was estimated by analysis of molecular variance (AMOVA) [30] using GenALEx 6.503 [31]. Significance of estimated variance components was assessed based on 10,000 random permutations. Calculation of number of different alleles (Na), number of effective alleles (Ne), Shannon’s information index (I), expected heterozygosity (He) and unbiased expected heterozygosity (uHe) were computed for these two hypothetical populations using ‘allele frequency data parameters’ option in GenALEx 6.503 [31].

## 3. Results

### 3.1 Indel marker profile in rice landraces of Chhattisgarh, India

Most of the InDel markers amplified two bands/alleles in the collection of examined landraces. The higher molecular weight band corresponds to insertion and the lower band corresponds to deletion allele for each marker. Due to the insertion and deletion nature of these InDel markers, all showed 100% polymorphism. Since we carried out pre-screening of polymorphism among Dongjinbyeo, Swarna and other six diverse landraces in capillary electrophoresis system, the band size of each marker was estimated. It was revealed that the insertion/deletion size of these markers ranged between 6 bp to 68 bp. The marker R3M37 amplified three bands of 194, 240 and 282 bp among the 192 genotypes (Table 1). It has been found that marker R1M37 and R7M20 were polymorphic between Dongjinbyeo and Swarna but they were monomorphic among other 190 landraces/genotypes. In contrast, R6M14, R11M23 and R12M27 were polymorphic among landraces but monomorphic between Dongjinbyeo and Swarna. The marker R1M37 showed dominant reaction (presence in Dongjinbyeo but absence in Swarna), while rest of the InDel markers were co-dominant in nature. Moreover, R7M20 showed heterozygous marker profile in all the tested landraces. Overall 42 InDel markers amplified 84 polymorphic bands in these rice genotypes.

In total, six InDel markers detected null alleles in 13 genotypes. Amplification of R2M10 has detected the highest null alleles in seven landraces namely, Pangudi goindi, Kapri, Alsenga, Gangabaru, Jonyaphool, Marandhan and Panwar. While, R4M50, R6M14, R6M44 and R10M30 marker generated null allele each in single genotype. Another marker R9M20 detected null alleles in three genotypes namely, Karhani, Ruchidhan and Sonkharcha. Of the 190 tested genotypes, 12 had generated null alleles in at least one of the tested InDel loci. The landrace, Gangabaru generated null alleles in two InDel marker loci, R2M10 and R6M44. Among 190 landraces/genotypes, 47 were detected with heterozygous band profile in one to seven loci. The landrace, Dowana had detected the highest proportion (16.7%) of heterozygous band profile. While, the lowest heterozygous band profile (2.4%) was detected in other 32 genotypes.

### 3.2. Informativeness of the InDel marker

A total of 10 rare alleles were identified from the amplification of 42 InDel loci. Of them, eight were low molecular weight alleles (resulted from deletion) and remaining two were higher molecular weight allele (resulted from insertion). These rare alleles were present only in 0.5% to 4.2% of the tested landraces. The polymorphic information content of these 42 markers ranged from 0.01 to 0.98. Seventeen markers had the ideal PIC values that were ranged between 0.35 and 0.50. The marker which amplified rare alleles had detected very low PIC values. The resolving power (R_p_) of these tested InDel markers varied from 0.01 to 1.86. Of the 42 markers, 14 had R_p_ value of more than 1.0. Effective multiplex ratio (EMR) of these markers ranged between 2.0 to 3.0. The highest EMR value was detected in R3M37. The marker index (MI) varied from 0.02 to 1.96. InDel markers, R3M37, R7M20 and R12M33 had MI values of more than 1.0 (Table 1).

### 3.3. Genetic differentiation of rice landraces based on ‘InDel Molecular Index’

*Indica* and *japonica* allele were assigned according to their amplification of InDel markers in Swarna and Dongjinbyeo, respectively. A total of 42 polymorphic InDel markers were used for differentiating these 188 rice landraces and two local varieties of Chhattisgarh, India into *indica* and *japonica* related genotypes based on ‘InDel molecular index’ as suggested by Lu et al. [8]. Results from the computation of ‘Indel molecular index’ indicated that 104 rice landraces were similar to ‘*indica*’ type. This ‘*indica*’ type also included three tested *indica* cultivars Swarna, Mahamaya and Rajeshwari. Another 60 landraces were placed under ‘close to *indica* type’ (Table 2). It was found that three rice landraces *i*.*e*. Kalajeera, Kapri and Tulsimala were of ‘close to *japonica*’ type and 21 landraces *i*.*e*. Laxmibhog, Tulsimongra, Jauphool, Rudra, Tulsibhog, Badshabhog-2, Gangabaru, Tulsi Manjari, Kadamphool, Lokti Musi, Govardhan Kali Kamod 2, Panwar, Badshabhog Selection-1, Korma, Bhaisapuchhi, Katrani-4, Katrani-7, Kondha Koya, Gomti, Maran Dhan and Santio were identified as intermediate type. Of these 21 intermediate type landraces, 18 landraces had more *japonica* specific alleles as compare to *indica* specific alleles which indicated that these varieties were closer to *japonica*. Three other rice landraces *i*.*e*. Gomti, Maran Dhan and Santio had more *indica* specific alleles (F_i_ ranged from 0.52 to 0.58) as compare to *japonica* type alleles. The result from the calculation of ‘InDel molecular index’ was also verified with AMOVA. Two groups/populations were made based on the calculated InDel molecular index. All the 21 ‘intermediate’ type, three ‘close to *japonica*’ genotypes and Dongjinbyeo were made a separate population (population 2) from rest of the landraces/genotypes for AMOVA study.

**Table 2:** Classification of rice landraces into *indica* or *japonica* types based on the ‘InDel Molecular Index’.

S.No.	Indica specific allele frequency (F_i_)	Japonica specific allele frequency (F_j_)	Type of rice identified by InDel Index	Total No.	Name of landraces
1	>0.90	<0.10	Typical indica	1	Swarna
2	0.75–0.89	0.11–0.25	Indica	106	Bathrash, Pratiksha, Bhadvel, Dhaura Mundariya, Safri, Dubraj, Agyasal, Jhimipras Samlayi, Dhaura Mundariya, Gangachur, Byalo, Bhusu, Sanchorma, Satra Safri, Dhaniyaphool, Lalbarhasal, Barhasal-2, Bhunduluchai, Barhasal-3, Khetganga, Bashabhog, Nariyalphool, Kanakbhog, Mahamaya, Rajeshwari, Hr 14–1 Heera, Matko Dhan, Indjopa, Ramigauri, Arokhutu, Hathi Panjra, Ramlaxman, Raja Banga, Kari Gilash, Muni Bhog, Sua Pankhi, Mala Gauri, Dokra Dokri, Nariyal Jhoba, Chhindmauri, Sugandha, Dandrice, Beedela Dhan, Sonagathi-2, Phalod Dhan, Baigani Dhan, Asam Chudi, Jana Dhan, Rela Dhan, Jhunuprash, Odha Dhan Banarsi, Lochai, Gadur Sela, Loindi, Godadani, Chatiya Nakhi, Bhatha Masri, Kanchan, Sutai Dhan, Bhujnin, Sadachar, Mahabaikoni, Bhejrimai Dhan, Ratan Chudi, Rani Parewa, Kari Alcha, Jhilli Safri, Turiya Khudig, Rang Chudi, Mota Chudi, Kharikha Kuchi, Memri Khedi, Samarlengda, Mayath, Mota Safri, Kalinga, Bhusu, Kabeli, Lalapana, Surmatiya, Manmohan, Jalgundi, Churlai Banko, Rani Kajar, Parwat Kal, Jhoomar, Asam Chudi-3, Jalkeshar, Sudama, Hajan, Ikkopatla, Haruna Masri, Kosawari, Bansgathi, Bhata Nakhi, Masri, Ikkomota, Gaurimala, Sichar, Rajabangla, Hardigathi, Ramlaxman, Luchai- 2, Maidubraj, Pancho, Bhata Masri-2
3	0.61–0.74	0.26–0.39	Close to indica	60	Anjani, Jonyaphool, Bhajna, Ratajhinga, Sawani, Jhimipras, Pangudi Goindi, Sihar, Bhusi, Karhani, Barhani, Kanakbans, Jhimipras-2, Alsenga, Ruchi Dhan, Lajini Super, Gudkamal Dhan, Nimaliya Banki, Jonyaphool, Brown Rice-1, Brown Rice-2, Dubraj, Modipeera, Petgadi, Sindursal, Bansveera Dhan, Lalma Dhan, Kareni Dhan, Parra Dhan, Ramshri, Danwar, Baiga Seeng, Mohlai Banko, Manki, Khajoor, Kumhdayin, Chhindmauri, Mejhri, Bodibaja, Jela, Anjaniya, Bakti Chudi, Nanded, Antarved, B.D. Safri-2, Ankapalli, Gatuvan, Baikoni, Chinni Paras, Jalsinga, Agni Fag, Bahal Binjo, Kari Grass, Asam Chudi-2, Hathi Pinjara, Safri-17, Ramjhilli, Majori, Dowana, Sonkharcha
4	0.40–0.60	0.40–0.60	Intermediate	21	Laxmibhog, Tulsimongra, Jauphool, Rudra, Tulsibhog, Badshabhog-2, Gangabaru, Tulsi Manjari, Kadamphool, Maran Dhan, Lokti Musi, Govardhan Kali Kamod 2, Santio, Panwar, Badshabhog Selection-1, Korma, Bhaisapuchhi, Gomti, Katrani-4, Katrani-7, Kondha Koya
5	0.26–0.39	0.61–0.74	Close to japonica	3	Kalajeera, Kapri, Tulsi Mala
6	0.11–0.25	0.75–0.89	Japonica	0	-
7	<0.10	>0.90	Typical japonica	1	Dongjinbyeo

Based on AMOVA it was detected that there was a significant genetic differentiation (ɸ_PT_ = 0.642, P = 0.000) between two populations. Total molecular variance was partitioned into two, of which, 64% explained variation among populations and remaining 36% explained variation within populations (Table 3). Result of this study was further confirmed by principle component analysis and cluster analysis based on binary data matrix of all InDel markers in the landraces. AMOVA analysis also detected contribution of 17 InDel loci which were responsible significantly for the above genetic differentiation. Overall the population 2 (‘*japonica*’ and ‘close to *japonica*’ landraces) revealed greater Shannon information index of 0.35±0.03, expected heterozygosity of 0.23±0.02 and unexpected heterozygosity of 0.23±0.02 compared to the ‘*indica*’ or ‘close to *indica*’ population (population 1) where Shannon information index, expected heterozygosity and unbiased expected heterozygosity were 0.32±0.03, 0.20±0.02 and 0.20±0.02, respectively.

**Table 3:** Analysis of molecular variance between proposed ‘indica’/’close to indica’ and ‘close to japonica’/’intermediate’ populations of rice landraces from Chhattisgarh, India.

Sources	Degree of freedom	Sum of Square	Mean Sum of Square	Estimated Variance	Proportion of Total Variance
Among population	1	614.09	614.09	13.94	64%
Within population	190	1476.66	7.77	7.77	36%
	191	2090.75		21.71	

### 3.4. Population structure and genetic diversity among rice landraces from Chhattisgarh, India

**Fig 3:**
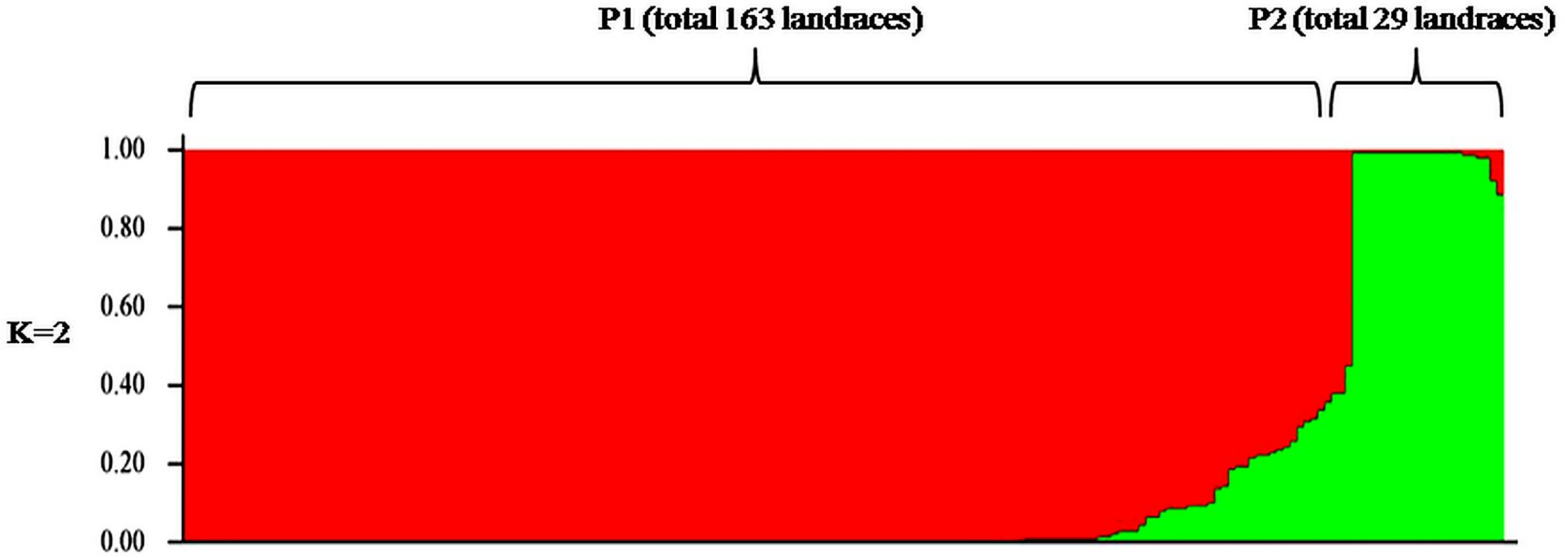
Population structure of traditional rice landraces of Chhattisgarh, India based on model-based clustering using STRUCTURE.

Both LnP(D) and Evano’s ΔK method revealed the presence of two genetically distinct clusters (K = 2; S1 Fig) among these traditional rice landraces in Chhattisgarh, India (Fig 3). Population 1 (P1) contained 163 landraces (85.1% of the total landraces) which had more *indica* alleles and categorized as ‘*indica*’ and ‘*close to indica*’ type as per InDel molecular index. These 163 landraces/genotypes also included typical *indica* cultivars Swarna, Mahamaya and Rajeswari. The other 29 landraces/genotypes (including Dongjinbyeo) (14.9% of the total landraces) were under population 2 (P2). Of these 29 landraces, three (Kalajeera, Kapri and Tulsimala) were ‘close to *japonica*’ type; 21 landraces were ‘intermediate’ type; remaining four landraces (Ruchidhan, Alsenga, Jonyaphool, Lalma Dhan) were ‘close to indica’ type according to InDel molecular index (Table 2). These two populations diverged at an allele frequency of 0.408, with an F_st_ (population-specific F_st_ calculated in STRUCTURE) of 0.676 and 0.580 for P1 and P2, respectively with an average F_st_ value of 0.627. From the above STRUCTURE analysis, 15 landraces/genotypes (7.8%) was detected as an admixture at K = 2.

**Fig 4:**
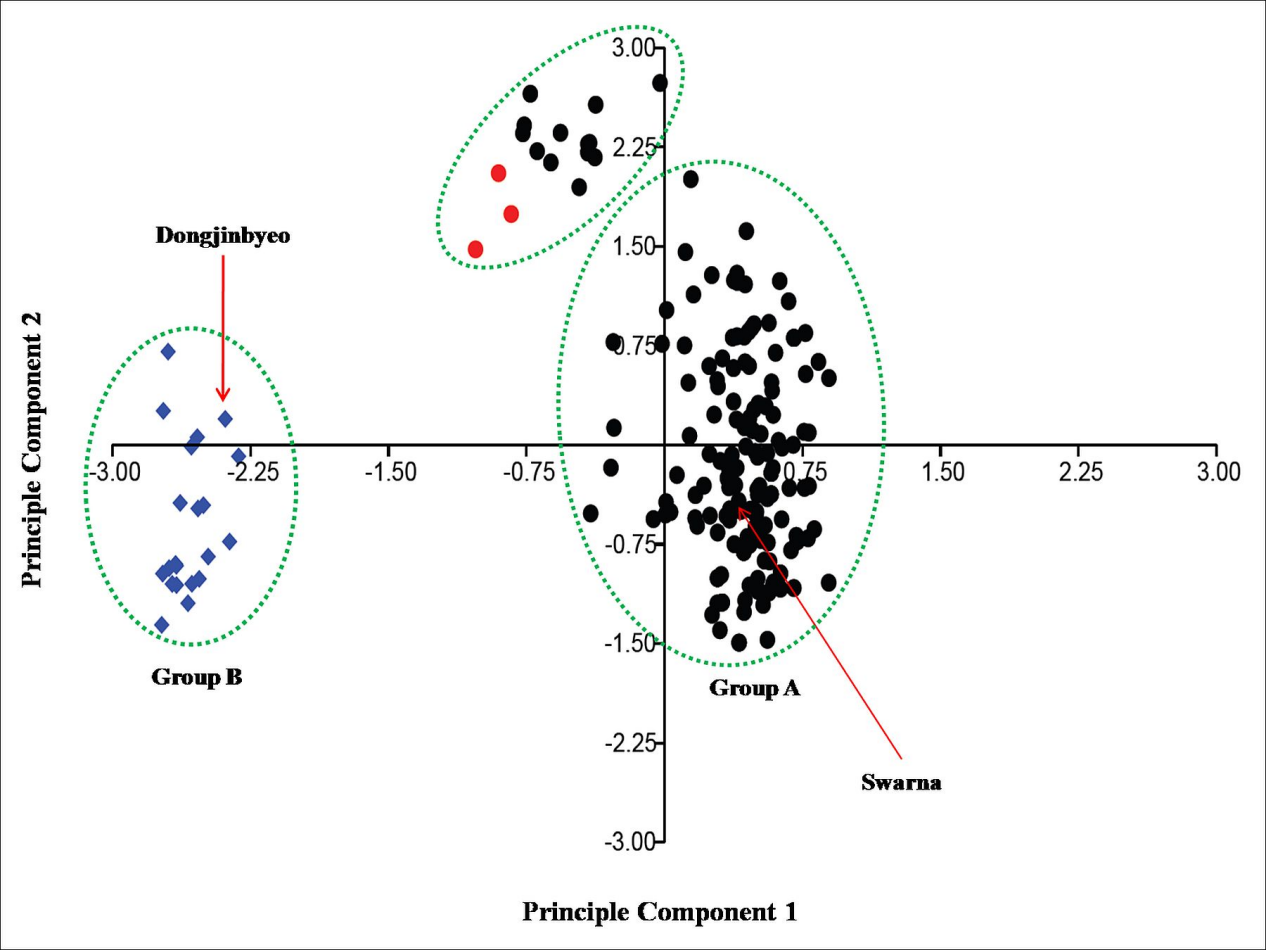
Genetic differentiation of 192 rice landraces/genotypes from Chhattisgarh, India based on principle component analysis. Note: Blue diamond represents rice genotypes that include Dongjinbyeo, ‘close to *japonica*’ and ‘intermediate’ type of landraces. Red circle represents remaining ‘intermediate’ type of landraces which have more *indica* allele. The black circle represents all the ‘*indica*’ and ‘close to *indica*’ type of rice landraces including Swarna, Mahamaya and Rajeshwari varieties.

A scatter plot was generated based on principle component analysis (PCA) of the binary data matrix developed from the 192 (including Dongjinbyeo and Swarna) rice genotypes. It demonstrated a clear pattern of two major groups differentiation (Fig 4). Spatial distance between genotypes in the scatter plot showed genetic relationships among the rice landraces. The first three principal components accounted for 48.60% of total variability in which first and second principal components explained for 34.02% and 7.88% of the total genetic variation, respectively. Noticeable genetic differentiation was observed between *indica* group and *japonica* group, as they were clustered into two major groups along the first principal component. Scatter diagram showed that all the ‘*indica* type’ and ‘close to *indica* type’ landraces were separated in group ‘A’ along with Swarna along the principle component 1 (PC1) axis. Whereas, all the ‘intermediate type’ landraces and ‘close to *japonica*’ type landraces were separated into group ‘B’ along with Dongjinbyeo along the PC1 and PC2 axes (Fig 4). Three rice landraces, namely Gomti, Marandhan and Santio were classified as intermediate type but they have more *indica* alleles as compare to *japonica*. Therefore, they were grouped with few ‘close to *indica***’** type landraces along the PC2 axis (Fig 4). The distribution of *indica* type landraces was more scattered than that of *japonica* type landraces in the scatter plot. PCoA for all the landraces based on the latitude, longitude and altitude of their collection districts revealed three main grouping according to their ecological regions. Chhattisgarh state is geographically distributed into three ecological regions *i*.*e*. Northern hills, Chhattisgarh plains and Bastar plateau. According to PCoA scatter diagram, all the collection sites/landraces were grouped into these three ecological regions (S2 Fig). Genotypes Kalajeera, Kapri, Tulsimala which comes under ‘close to *japonica*’ types in this study were collected from Ambikapur district of Chhattisgarh. The altitude of Ambikapur is high (603.00 meters) and it belongs to Northern hill region. All the ‘intermediate’ type genotypes were collected from different districts of Chhattisgarh plains where altitude ranges from 200 m to 400 m. The remaining close to *indica* type genotypes was collected from various districts and they did not show specific pattern of distribution.

**Fig 5:**
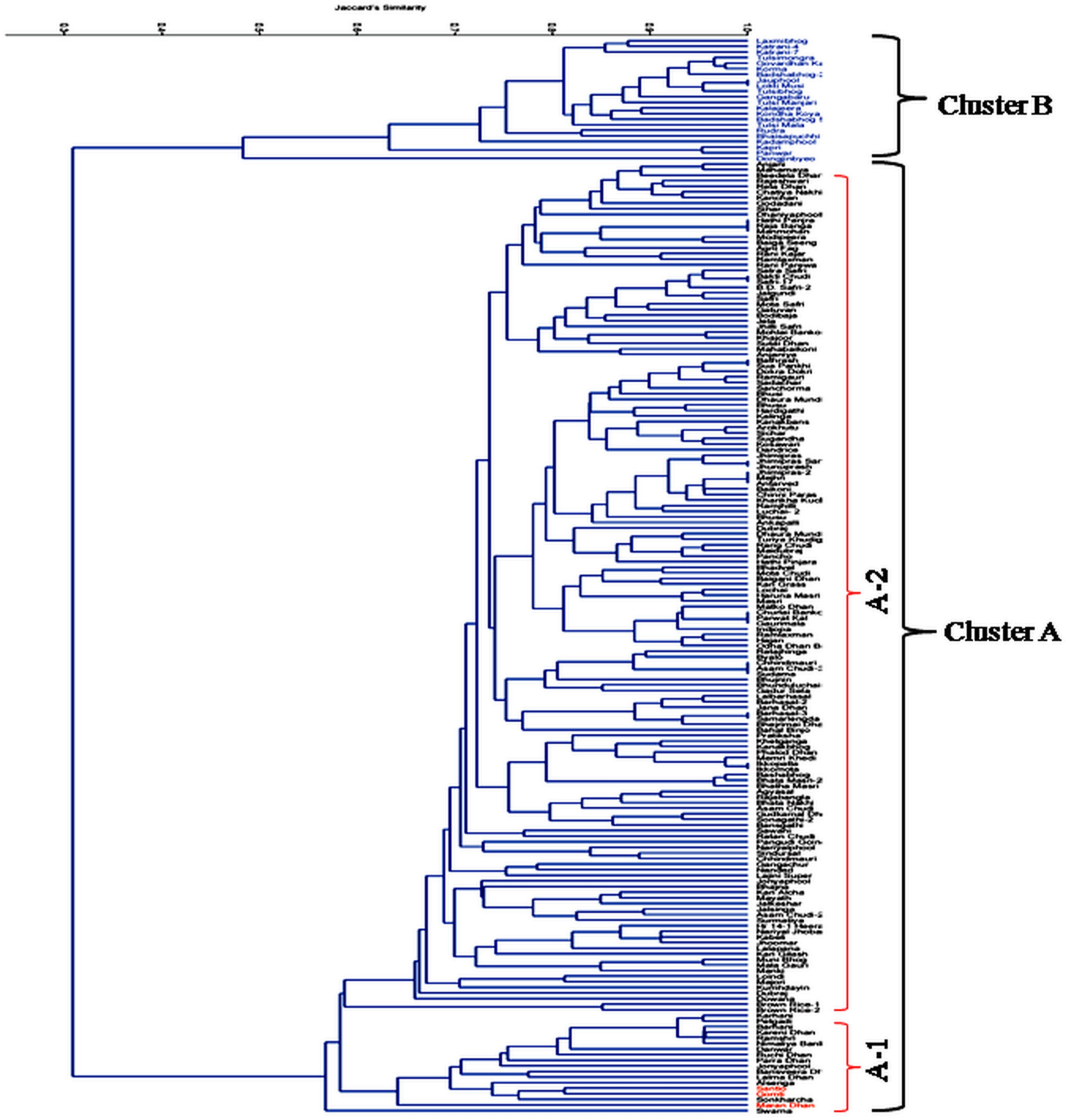
Dendrogram depicting the genetic diversity of 190 rice landraces/genotypes from Chhattisgarh, India based on cluster analysis. Note: Blue colour represents rice genotypes that include Dongjinbyeo, ‘close to *japonica*’ and ‘intermediate’ type of landraces. Red colour represents remaining ‘intermediate’ type of landraces which have more *indica* allele. The black colour represents all the ‘*indica*’ and ‘close to *indica*’ type of rice landraces including Swarna, Mahamaya and Rajeshwari varieties.

Similarly, UPGMA cluster analysis based on Jaccard’s similarity coefficient had detected a dendrogram that had 0.94 cophenetic correlation with the pairwise genetic distance. Pairwise genetic distance based on Jaccard’s similarity varied from 3.75% to 100%. The lowest similarity was detected between Dongjinbyeo and Swarna. The highest genetic similarity (100%) was detected between Jauphool and Lokti Mushi in ‘intermediate’ InDel molecular index group. Similarly 22 other landraces in ‘*indica*’ or ‘close to *indica*’ group also showed 100% Jaccard’s similarity among the pair of genotypes. Cluster analysis followed the pattern of PCA analysis. All the 192 rice genotypes were grouped into 2 major clusters ‘A’ and ‘B’ at 30% Jaccard’s similarity level (Fig 5). The cluster ‘A’ grouped 170 genotypes and cluster ‘B’ grouped 22 genotypes. Cluster ‘A’ contained the reference *indica* genotype, Swarna. In contrast, cluster ‘B’ included the reference *japonica* genotype Dongjinbyeo. All the 22 genotypes/landraces (including Dongjinbyeo) in cluster ‘B’ were classified as ‘close to *japonica*’ based on ‘InDel molecular index’. Cluster ‘A’ again subdivided into two sub-clusters at 60% Jaccard’s similarity level. The sub-cluster A1 included 17 landraces that had Marandhan, Santio and Gomti. These three landraces was classified as intermediate type based on InDel molecular index. Moreover, these three landraces have more *indica* specific alleles like in ‘close to *indica*’ or *indica* type. Subcluster A2 consisted of 153 rice landraces/genotypes which were revealed to be *indica* type based on InDel molecular index (Fig 5).

## 4. Discussion

Large number of landraces was collected from different tribal parts of Chhattisgarh, India for conservation and varietal protection. These landraces were maintained at Indira Gandhi Krishi Viswavidyalaya, Raipur (CG), India. After collection, these genotypes were evaluated in experimental field and redundant accessions were curtailed based on morphological observations on plants and grains. Such of 190 landraces/genotypes were used in the present study to decipher genetic diversity and population structure based on InDel markers. These markers are highly reliable and informative like SSR but they are biallelic compare to multiallelic in SSR. InDel markers have been successfully used for genetic diversity studies in rice [4,12,32,33,34,35,36], wheat [37], chickpea [38] and *Arabidopsis* [39,40]. These markers are preferred over other markers due to their codominant nature, cost-effectivity, and easy to resolve on agarose gel. Due to this simplicity in electrophoretic separation, we have chosen this group of markers for rapid determination of population structure and genetic diversity in this collection of traditional rice landraces. All the InDel markers revealed a clear and consistent band profile with InDel size as reported by Lu et al. [8] and Shen et al. [9]. With repeated PCR reactions, we confirmed the null allele amplifications of six markers in 13 genotypes. These null alleles were arisen probably due to the mutations in the primer binding sites of the genomic DNA of respective landraces [41]. Further, these InDel markers have detected heterozygous banding profile in 47 genotypes/landraces to the extent of 2.4% to 16.7%. Amplification of heterozygous bands was also reported in wild species of rice [12].

Lu et al. [8] used InDel markers for generating ‘InDel molecular index’ which was exploited for differentiating *indica* and *japonica* type as proposed initially by Shen et al. [9]. Moreover, these markers were also used for genotyping of wild rice species [12] and detecting difference in basmati and non-basmati rice genotypes [11]. In the present study 42 InDel markers which were distributed across the genome of *Oryza sativa*, were used to differentiate the rice landraces into different category according to Lu et al. [8]. Three landraces were found ‘close to *japonica*’ category due to their more proportion of *japonica* related alleles. Further these three landraces were adapted into the higher altitude (603 m) of Ambikapur district of Chhattisgarh state in India. Further 21 other landraces were classified as ‘intermediate’ category. Among the 21 ‘intermediate’ types of landraces, three genotypes, Marandhan, Santio and Gomti were distinct from rest as they contained more *indica* alleles. These three landraces were grouped with ‘*indica*’ or ‘close to *indica*’ category of landraces in both PCA diagram and cluster analysis (Fig 4 and Fig 5). All the three ‘close to *japonica*’ landraces (Kalajeera, Kapri and Tulsimala) and remaining 18 ‘intermediate’ landraces were grouped in a separate cluster in both PCA and cluster analysis. Further this population differentiation was also tested in STRUCTURE where two clear populations were found and P2 contained all the ‘close to *japonica*’ and ‘intermediate’ type of landraces (Fig 3; Table 2). The differentiation between *indica* and *japonica* subspecies was the result of adaptive evolution which resulted from accumulation of genetic variation across the rice genome during domestication and selection by humans in various ecological habitats [42]. It has been well recognized that a variation in DNA sequences such as DNA rearrangement, base substitution, insertion and deletion was the fundamental cause of *indica japonica* differentiation [43]. Moreover origins of different types of InDels in these landraces could be created through the mobilization of transposon and retroelement [44]. This genetic differentiation between *indica*/close to *indica* and close to *japonica* /intermediate was further confirmed by the significant ɸ_PT_ value obtained from AMOVA.

Till now there was a perception that all the rice varieties grown in Chhattisgarh, India are of *indica* type but the result in the study indicated that some of the indigenous rice landraces may have *japonica* and intermediate type features. These *japonica* and intermediate type landraces might evolve from *japonica* related parents or from wild rice accessions with *japonica* type alleles. These landraces are being evaluated thoroughly for morphological, biochemical and molecular level. In future, the categorization of some of these landraces as ‘close to *japonica*’ and/or ‘intermediate’ will be verified with hierarchical grouping involving multi-trait analysis. Result showed that the reference *indica* variety Swarna comes under typical *indica* type whereas reference *japonica* variety Dongjinbyeo comes under typical *japonica* type. The very genetic diversity between these two categories can be exploited for rice breeding in future. Thus ‘InDel molecular index’ along with the agronomic features of these landraces will be considered in future to harness their recombinogenic transgressive segregates as high yielding selection or to develop hybrid rice [4]. As these landraces are adapted to climatic condition of Chhattisgarh states for several hundred years, it is important to exploit useful genetic diversity for rice breeding according to the need of local tribals and market requirement for high yield parameters. These *indica* and *japonica* type landraces will be used to develop hybrids towards utilization of *indica* x *japonica* inter sub-specific heterosis [45]. The landraces in the ‘close to *japonica*’/‘intermediate’ category can be utilized in hybridization with ‘*indica*’ category for exploiting their hybrid vigour or transgressive segregation. This hybridization might avoid the so-called sterility problem in *indica*-*japonica* inter-subspecies hybridization.

## 5. Conclusion

Findings from the present experiment has its important implication in rice genetic improvement by selecting diverse and significantly differentiated ‘*indica*’ or ‘close to *japonica*’ category of rice landraces in breeding programs. In addition, utilization of strong hybrid vigour (heterosis) derived from *indica*–*japonica* hybrids can promote the production of ‘super hybrid rice’ in this locally adapted landraces. This collection of landraces will be further evaluated for several biotic and abiotic stress tolerances. Based on the examined stress tolerance, trait based-breeding program will be taken for genetic improvement of these landraces in Chhattisgarh, India. The co-dominant InDel markers used in this study has immense scope to use them for trait mapping, association mapping and marker assisted selection during the exploitation of these landraces in rice breeding program.

## Supporting information

S1 Fig **Plotting of LnP(D) [a] and ΔK [b] against number of cluster (K) from the STRUCTURE simulation summary**.
(TIF)

S2 Fig Scatter diagram of 190 rice landraces based on geographical information (latitude, longitude and altitude) on collection sites in Chhattisgrah, India. (TIF)

S1 Table Details of rice landraces and varieties used for this study. (DOCX)

## References

[1] FAO. Rice market monitor. 2017; 20 (1): 1–38. Data accessed on 15 May, 2017. Available at http://www.fao.org/fileadmin/templates/est/COMM_MARKETS_MONITORING/Rice/Images/RMM/RMM_APR17_H.pdf.

[2] Morishima H. Wild plants and domestication In: Tsunoda S, Takahashi N (eds) Biology of rice. Elsevier, Amsterdam 1984; 3–30.

[3] Brar DS, Khush GS. Utilization of wild species of genus *Oryza* in rice improvement In: Nanda JS, Sharma SD (eds) Monograph on genus *Oryza*. Plymouth; Science Publishers: Enfield, UK, 2003; pp. 283–309.

[4] Xiong Z, Zhang S, Wang Y, Brian V, Lloyd F, Tu M et al Differentiation and distribution of *indica* and *japonica* rice varieties along the altitude gradients in Yunnan Province of China as revealed by InDel molecular markers. Genetic Resources and Crop Evolution. 2010; 57: 891–902.

[5] Khush GS. Green revolution: the way forward. Nature Review Genetics. 2001; 2: 815–22. 10.1038/35093585 11584298

[6] Peng SB, Laza RC, Visperas RM, Khush GS, Virk P, Zhu D. Rice: progress in breaking the yield ceiling. In: Proceedings of the fourth International Crop Science Congress, September 26–October 1, 2004; Brisbane, Australia, Published on CD.

[7] Cheng KS, Wang XK, Lu YX. The synthetical research and utilization of Yunnan rice resource: the reorganization of the Asian cultivated rice classification. Acta Agronomica Sinica. 1984; 10: 271–279.

[8] Lu BR, Cai XX, Jin X. Efficient *indica* and *japonica* rice identification based on InDel molecular method: its implication in rice breeding and evolutionary research. Progress in Natural Sciences. 2009; 19: 1241–1252.

[9] Shen YJ, Jiang H, Jin JP, Zhang ZB, Xi B, He YY et al Development of genome-wide DNA polymorphism database for map based cloning of rice genes. Plant Physiology. 2004; 135: 1198–1205. doi: 10.1104/pp.103.038463 15266053 10.1104/pp.103.038463 PMC519040

[10] Xingxing C, Jing L, Yinqiu Q, Wei Z, Zhiping S, Baorong L. Differentiation of Indica-Japonica rice revealed by insertion/deletion (InDel) fragments obtained from the comparative genomic study of DNA sequences between 93–11 (*Indica*) and Nipponbare (*Japonica*). Frontiers of Biology in China. 2007; 2(3): 291–296.

[11] Steele KA, Ogden R, McEwing R, Briggs H, Gorham J. InDel markers distinguish Basmatis from other fragrant rice varieties. Field Crops Research. 2008; 105 (1–2): 81–87.

[12] Niihama M, Mochizuki M, Kurata N, Nonomura KI. PCR-based InDel markers co-dominant between *Oryza sativa*, *japonica* cultivars and closely-related wild *Oryza species*. Breeding Science. 2015; 65: 357–361. doi: 10.1270/jsbbs.65.357 26366120 10.1270/jsbbs.65.357 PMC4542938

[13] Yamaki S, Ohyanagi H, Yamasaki M, Eiguchi M, Miyabayashi T, Kubo T et al Development of InDel markers to discriminate all genome types rapidly in the genus *Oryza*. Breeding Science. 2013; 63: 246–254. doi: 10.1270/jsbbs.63.246 24273419 10.1270/jsbbs.63.246 PMC3770551

[14] Das B, Sengupta S, Parida SK, Roy B, Ghose M, Prasad M et al Genetic diversity and population structure of rice landraces from Eastern and North Eastern States of India. BMC Genetics. 2013; 14: 71 doi: 10.1186/1471-2156-14-71 23945062 10.1186/1471-2156-14-71 PMC3765237

[15] Choudhury DR, Singh N, Singh AK, Kumar S, Srinivasan K, Tyagi RK et al Analysis of genetic diversity and population structure of rice germplasm from north-eastern region of India and development of a core germplasm set. PLoS ONE. 2014; 9(11): e113094 doi: 10.1371/journal.pone.0113094 25412256 10.1371/journal.pone.0113094 PMC4239046

[16] Roy S, Banerjee A, Mawkhlieng B, Misra AK, Pattanayak A, Harish GD et al Genetic diversity and population structure in aromatic and quality rice (*Oryza sativa* L.) landraces from North Eastern India. PLoS ONE. 2015; 10(6): e0129607 doi: 10.1371/journal.pone.0129607 26067999 10.1371/journal.pone.0129607 PMC4467088

[17] Richharia RH. An aspect of genetic diversity in rice. Oryza.1979; 16(1): 1–31.

[18] Panigrahi P. Genetic divergence analysis in indigenous landraces of Bastar region in rice (Oryza sativa L.). A M.Sc. (Ag) thesis, Department of Genetics and Plant Breeding, College of Agriculture, Faculty of Agriculture, Indira Gandhi Krishi Viswavidyalaya, Raipur, Chhattisgarh, India. 2016; 177. Available from; https://www.google.co.in/?gfe_rd=cr&ei=lpgaWfTONObH8geqvYKADQ#q=praveen+panigrahi+thesis

[19] Fukuoka S, Suu TD, Ebanna K, Trinh L. Diversity in phenotypic profiles in landraces populations of Vietnamese rice: a case study of agronomic characters for conserving crop genetic diversity on farm. Genetic Resources and Crop Evolution. 2006; 53: 753–761.

[20] Frankel OH, Soule ME. Conservation and Evolution. New York, USA: Cambridge University Press; 1981.

[21] Kottapalli KR, Narasu ML, Jena KK. Effective strategy for pyramiding three bacterial blight resistance genes into fine grain rice cultivar, Samba Mahsuri, using sequence tagged site markers. Biotechnology Letter. 2010; 32: 989–996. 10.1007/s10529-010-0249-1 20349335

[22] Pritchard JK, Stephens M, Donnelly P. Inference of population structure using multilocus genotype data. Genetics. 2000; 155: 945–959. 10835412 10.1093/genetics/155.2.945 PMC1461096

[23] Falush D, Stephens M, Pritchard JK. Inference of population structure using multilocus genotype data: linked loci and correlated allele frequencies. Genetics. 2003; 164: 1567–1587. 12930761 10.1093/genetics/164.4.1567 PMC1462648

[24] Evano G, Regnaut S, Goudet J. Detecting the number of clusters of individuals using the software STRUCTURE: a simulation study. Molecular Ecology. 2005; 14: 2611–2620. doi: 10.1111/j.1365-294X.2005.02553.x 15969739 10.1111/j.1365-294X.2005.02553.x

[25] Hammer Q, Harper DAT, Ryan PD. PAST: Paleontological statistics software package for education and data analysis. Palaeontologia Electronica. 2001; 4(1): 9.

[26] Powell W, Morgante M, Andre C, Hanafey M, Vogel J, Tingey S et al The comparison of RFLP, RAPD, AFLP and SSR (microsatellite) markers for germplasm analysis. Molecular Breeding. 1996; 2: 225–238.

[27] Milbourne D, Meyer R, Bradshaw JE, Baird E, Bonar N, Provan J et al Comparison of PCR-based marker systems for the analysis of genetic relationships in cultivated potato. Molecular Breeding. 1997; 3: 127–136.

[28] Prevost A, Wilkinson MJ. A new system of comparing PCR primers applied to ISSR fingerprinting of potato cultivars. Theoretical and Applied Genetics. 1999; 98: 107–112.

[29] Jain S, Jain RK, McCouch SR. Genetic analysis of Indian aromatic and quality rice (*Oryza sativa* L.) germplasm using panels of fluorescently labelled microsatellite markers. Theoretical and Applied Genetics. 2004; 109: 965–977. doi: 10.1007/s00122-004-1700-2 15309297 10.1007/s00122-004-1700-2

[30] Excoffier L, Smouse PE, Quattro JM. Analysis of molecular variance inferred from metric distances among DNA haplotypes: application to human mitochondrial DNA restriction sites. Genetics. 1992; 131: 479–491. 1644282 10.1093/genetics/131.2.479 PMC1205020

[31] Smouse PE, Whitehead MR, Peakall R. An informational diversity framework, illustrated with sexually deceptive orchids in early stages of speciation. Molecular Ecology Resources. 2015; 15(6): 1375–1386. doi: 10.1111/1755-0998.12422 25916981 10.1111/1755-0998.12422

[32] Hayashi K, Yoshida H, Ashikawa I. Development of PCR-based allele-specific and InDel marker sets for nine rice blast resistance genes. Theoretical and Applied Genetics. 2006; 113: 251–260. doi: 10.1007/s00122-006-0290-6 16791691 10.1007/s00122-006-0290-6

[33] Liu P, Cai XX, Lu BR. Single-seeded InDel fingerprints in rice: an effective tool for indica-japonica rice classification and evolutionary studies. Journal of Systematics and Evolution. 2012; 50: 1–11.

[34] Wu DH, Wu HP, Wang CS, Tseng HY, Hwu KK. Genome-wide InDel marker system for application in rice breeding and mapping studies. Euphytica. 2013; 192: 131–143.

[35] Zeng YX, Wen ZH, Ma LY, Ji ZJ, Li XM, Yang CD. Development of 1047 insertion-deletion markers for rice genetic studies and breeding. Genetics and Molecular Research. 2013; 12: 5226–5235. doi: 10.4238/2013.October.30.7 24301783 10.4238/2013.October.30.7

[36] Yonemaru JI, Choi SH, Sakai H, Ando T, Shomuru A, Yano M et al Genome-wide Indel markers shared by diverse Asian rice cultivars compared to Japanese rice cultivar ‘Koshihikari’. Breeding Science. 2015; 65: 249–256. doi: 10.1270/jsbbs.65.249 26175622 10.1270/jsbbs.65.249 PMC4482175

[37] Raman H, Raman R, Wood R, Martin P. Repetitive InDel markers within the ALMT1 gene conditioning aluminium tolerance in wheat (*Triticum aestivum* L.). Molecular Breeding. 2006; 18: 171–183.

[38] Das S, Upadhyaya HD, Srivastava R, Bajaj D, Gowda CLL, Sharma S et al Genome-wide insertion–deletion (InDel) marker discovery and genotyping for genomics-assisted breeding applications in chickpea. DNA Research. 2015; 22(5): 377–386. doi: 10.1093/dnares/dsv020 26385353 10.1093/dnares/dsv020 PMC4596403

[39] Hou XH, Li LC, Peng ZY, Wei BY, Tang SJ, Ding MY et al A platform of high-density INDEL/ CAPS markers for map-based cloning in Arabidopsis. Plant Journal. 2010; 63: 880–888. doi: 10.1111/j.1365-313X.2010.04277.x 20561258 10.1111/j.1365-313X.2010.04277.x

[40] Pacurar DI, Pacurar ML, Street N, Bussell JD, Pop TI, Gutierrez L et al A collection of INDEL markers for map-based cloning in seven Arabidopsis accessions. Journal of Experimental Botany. 2012; 63: 2491–2501. doi: 10.1093/jxb/err422 22282537 10.1093/jxb/err422 PMC3346218

[41] Callen DF, Thompson AD, Shen Y, Phillips HA, Richards RI, Mulley JC et al Incidence and origin of null alleles in the (AC)n microsatellite markers. American Journal of Human Genetics. 1993; 52: 922–927. 8488841 PMC1682051

[42] Sun J, Qian Q, Ma DR, Xu ZJ, Liu D, Du HB et al Introgression and selection shaping the genome and adaptive loci of weedy rice in northern China. New Phytologist. 2013; 197: 290–299. doi: 10.1111/nph.12012 23106357 10.1111/nph.12012

[43] Wang J, Yu J, Wang J. Systematical research of rice genome based on the whole genome sequence. World Sci-tech R&D. 2003; 25(6): 35–41 (in Chinese). doi: 10.1038/s41598-017-02157-6

[44] Edwards JD, Lee VM, McCouch SR. Sources and predictors of resolvable InDel polymorphism assessed using rice as a model. Molecular Genetics and Genomics. 2004; 271: 298–307. doi: 10.1007/s00438-004-0979-7 14758543 10.1007/s00438-004-0979-7

[45] Sharma D, Sanghera GS, Sahu P, Sahu P, Parikh M, Sharma B et al Tailoring rice plants for sustainable yield through ideotype breeding and physiological interventions. African Journal of Agriculture Research. 2013; 8(40): 5004–5019.

