# Supplementary figures and images for: InDel marker based genetic differentiation and genetic diversity in traditional rice (Oryza sativa L.) landraces of Chhattisgarh, India
Source: PLoS One. 2017 Nov 30;12(11):e0188864. doi: 10.1371/journal.pone.0188864 (PMC5708757; doi:10.1371/journal.pone.0188864)

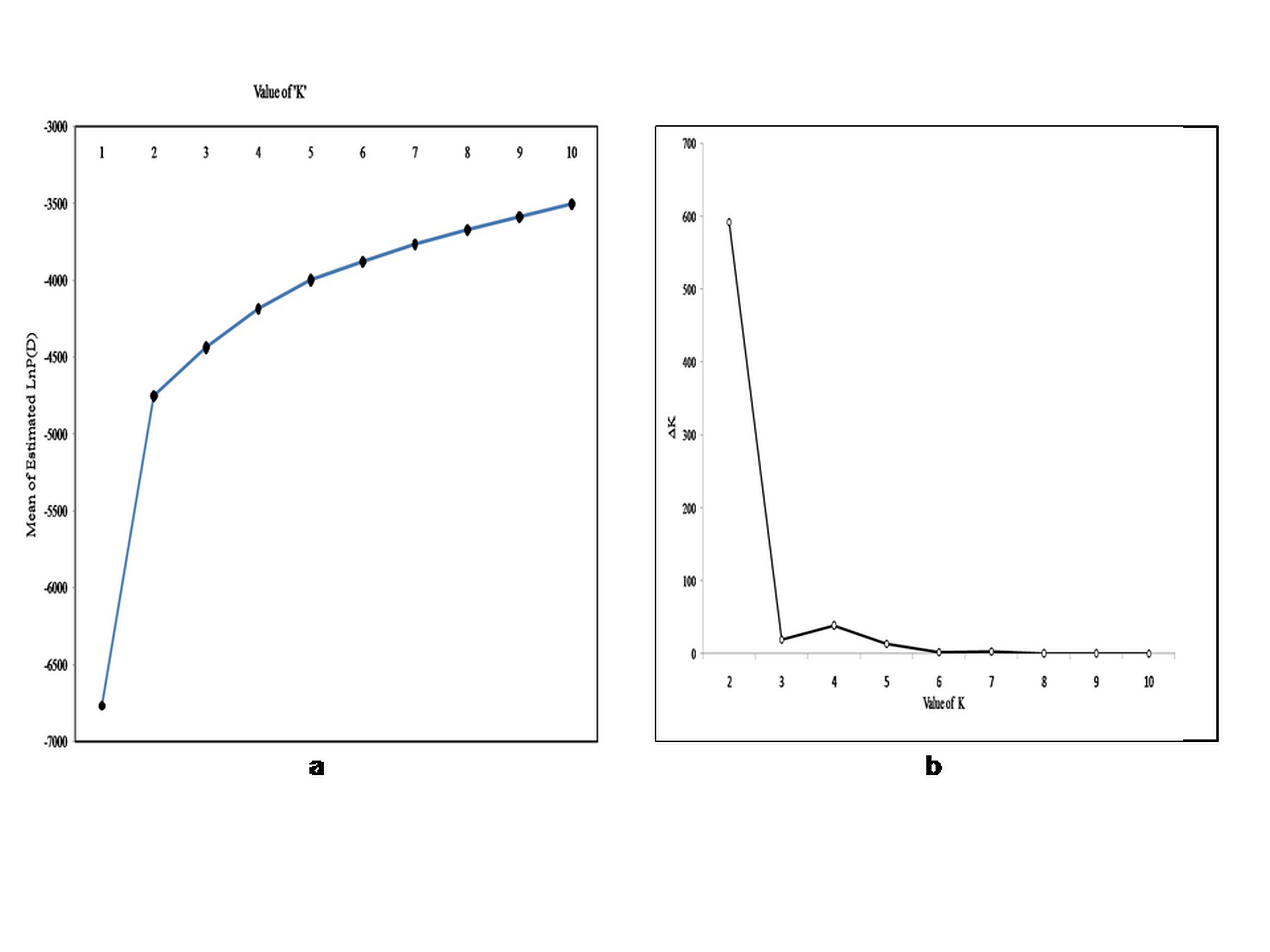

Supplement: S1 Fig — Plotting of LnP(D) [a] and ΔK [b] against number of cluster (K) from the STRUCTURE simulation summary. (TIF) [file pone.0188864.s001.tif]

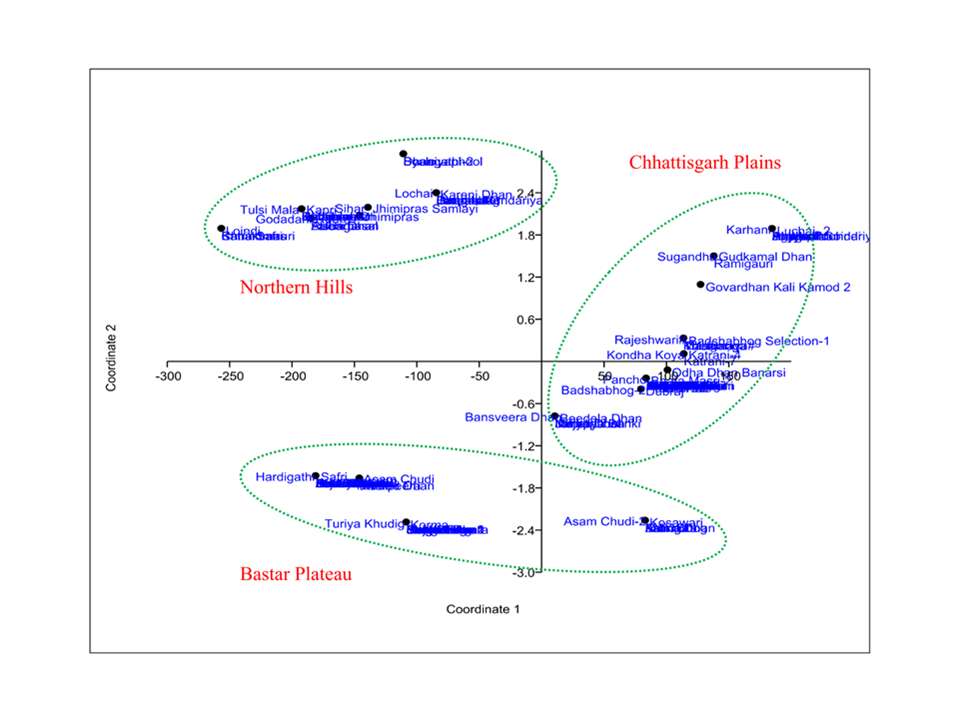

Supplement: S2 Fig — (TIF) [file pone.0188864.s002.tif]
