# Supplementary material for: InDel marker based genetic differentiation and genetic diversity in traditional rice (Oryza sativa L.) landraces of Chhattisgarh, India
Source: PLoS One. 2017 Nov 30;12(11):e0188864. doi: 10.1371/journal.pone.0188864 (PMC5708757; doi:10.1371/journal.pone.0188864)
Supplement: S1 Table — (DOCX) [file pone.0188864.s003.docx]

**Table S1: List of rice landraces used in this study with their geographical coordinates**

| **S.N.** | **Genotype name** | **IGKV ID** | **BARC ID** | **Collection Sites** | **Latitude** | **Longitude** | **Altitude** |
| --- | --- | --- | --- | --- | --- | --- | --- |
| 1 | Dongjinbyeo (*Japonica* cultivar) |  | 1 | Advanced Radiation Technology Institute, KAERI, Republic of Korea | 37.532600 | 127.024612 | 21.00 |
| 2 | Swarna (*Indica* cultivar) |  | 2 | Indira Gandhi KrishiVishwavidyalaya, Raipur (Chhattisgarh) India | 21.2513844 | 81.6296413 | 296.98 |
| 3 | Anjani | 16 | 6 | Raigarh | 21.8974003 | 83.3949632 | 225.99 |
| 4 | Bathrash | 19 | 7 | Raigarh | 21.8974003 | 83.3949632 | 225.99 |
| 5 | Jonyaphool | 23 | 9 | Raigarh | 21.8974003 | 83.3949632 | 225.99 |
| 6 | Pratiksha | 24 | 10 | Raigarh | 21.8974003 | 83.3949632 | 225.99 |
| 7 | Bhadvel | 25 | 11 | Raigarh | 21.8974003 | 83.3949632 | 225.99 |
| 8 | Bhajna | 27 | 13 | Raigarh | 21.8974003 | 83.3949632 | 225.99 |
| 9 | Ratajhinga | 28 | 14 | Ambikapur | 23.116444 | 83.196121 | 603.00 |
| 10 | Laxmibhog | 32 | 16 | Jashpur | 22.78745 | 83.847301 | 495.00 |
| 11 | Sawani | 34 | 17 | Dhamtari | 20.7015 | 81.554158 | 326.93 |
| 12 | Tulsimongra | 37 | 19 | Dhamtari | 20.7015 | 81.554158 | 326.93 |
| 13 | DhauraMundariya | 38 | 20 | Jashpur | 22.78745 | 83.847301 | 495.00 |
| 14 | Jhimipras | 41 | 21 | Baikunthpur | 23.271349 | 82.556456 | 556.53 |
| 15 | PangudiGoindi | 43 | 22 | Raigarh | 21.8974003 | 83.3949632 | 225.99 |
| 16 | Safri | 44 | 23 | Kondagaon | 19.595851 | 81.663777 | 591.72 |
| 17 | Dubraj | 46 | 25 | Dhamtari | 20.7015 | 81.554158 | 326.93 |
| 18 | Agyasal | 47 | 26 | Raigarh | 21.8974003 | 83.3949632 | 225.99 |
| 19 | Jauphool | 50 | 27 | Jashpur | 22.78745 | 83.847301 | 495.00 |
| 20 | Kalajeera | 53 | 28 | Ambikapur | 23.116444 | 83.196121 | 603.00 |
| 21 | Sihar | 66 | 33 | Surajpur | 23.214815 | 82.869446 | 549.83 |
| 22 | JhimiprasSamlayi | 67 | 34 | Surajpur | 23.214815 | 82.869446 | 549.83 |
| 23 | Kapri | 71 | 36 | Ambikapur | 23.116444 | 83.196121 | 603.00 |
| 24 | Bhusi | 72 | 37 | Kondagaon | 19.595851 | 81.663777 | 591.72 |
| 25 | DhauraMundariya | 74 | 38 | Raigarh | 21.8974003 | 83.3949632 | 225.99 |
| 26 | Gangachur | 77 | 39 | Jashpur | 22.78745 | 83.847301 | 495.00 |
| 27 | Karhani | 80 | 40 | Raigarh | 21.8974003 | 83.3949632 | 225.99 |
| 28 | Byalo | 82 | 41 | Balrampur | 23.611174 | 83.610896 | 521.45 |
| 29 | Bhusu | 83 | 42 | Dhamtari | 20.7015 | 81.554158 | 326.93 |
| 30 | Sanchorma | 99 | 44 | Ambikapur | 23.116444 | 83.196121 | 603.00 |
| 31 | SatraSafri | 101 | 45 | Koria | 23.38755 | 82.388578 | 667.25 |
| 32 | Barhani | 105 | 46 | Jashpur | 22.78745 | 83.847301 | 495.00 |
| 33 | Kanakbans | 111 | 47 | Koria | 23.38755 | 82.388578 | 667.25 |
| 34 | Jhimipras-2 | 112 | 48 | Ambikapur | 23.116444 | 83.196121 | 603.00 |
| 35 | Dhaniyaphool | 116 | 49 | Balrampur | 23.611174 | 83.610896 | 521.45 |
| 36 | Lalbarhasal | 117 | 50 | Sitapur | 22.77927 | 83.491024 | 595.41 |
| 37 | Alsenga | 118 | 51 | Sitapur | 22.77927 | 83.491024 | 595.41 |
| 38 | Barhasal-2 | 120 | 52 | Jashpur | 22.78745 | 83.847301 | 495.00 |
| 39 | RuchiDhan | 127 | 53 | Sitapur | 22.77927 | 83.491024 | 595.41 |
| 40 | Rudra | 129 | 54 | Sitapur | 22.77927 | 83.491024 | 595.41 |
| 41 | Bhunduluchai | 131 | 55 | Kondagaon | 19.595851 | 81.663777 | 591.72 |
| 42 | Barhasal-3 | 136 | 56 | Ambikapur | 23.116444 | 83.196121 | 603.00 |
| 43 | Khetganga | 146 | 57 | Raipur | 21.2513844 | 81.6296413 | 296.98 |
| 44 | Bashabhog | 148 | 58 | Dantewada | 18.84563 | 81.383933 | 519.14 |
| 45 | Tulsibhog | 149 | 59 | Raipur | 21.2513844 | 81.6296413 | 296.98 |
| 46 | Nariyalphool | 153 | 60 | Dhamtari | 20.7015 | 81.554158 | 326.93 |
| 47 | Badshabhog-2 | 154 | 61 | Balod | 20.731132 | 81.202306 | 331.00 |
| 48 | Lajini Super | 155 | 62 | Kondagaon | 19.595851 | 81.663777 | 591.72 |
| 49 | Gangabaru | 156 | 63 | Dantewada | 18.84563 | 81.383933 | 519.14 |
| 50 | GudkamalDhan | 158 | 64 | Janjgir | 22.0105407 | 82.5726805 | 272.65 |
| 51 | TulsiManjari | 162 | 65 | Dhamtari | 20.7015 | 81.554158 | 326.93 |
| 52 | Kanakbhog | 168 | 66 | Dhamtari | 20.7015 | 81.554158 | 326.93 |
| 53 | Mahamaya# | 173 | 68 | Raipur | 21.2513844 | 81.6296413 | 296.98 |
| 54 | Rajeshwari# | 174 | 69 | Raipur | 21.2513844 | 81.6296413 | 296.98 |
| 55 | Hr 14-1 Heera | 177 | 70 | Dantewada | 18.84563 | 81.383933 | 519.14 |
| 56 | MatkoDhan | 179 | 71 | Kanker | 20.272713 | 81.48831 | 400.05 |
| 57 | NimaliyaBanki | 180 | 72 | Kanker | 20.272713 | 81.48831 | 400.05 |
| 58 | Jonyaphool | 184 | 73 | Kanker | 20.272713 | 81.48831 | 400.05 |
| 59 | Kadamphool | 185 | 74 | Kondagaon | 19.595851 | 81.663777 | 591.72 |
| 60 | Indjopa | 186 | 75 | Kanker | 20.272713 | 81.48831 | 400.05 |
| 61 | Ramigauri | 192 | 76 | Janjgir | 22.0105407 | 82.5726805 | 272.65 |
| 62 | Brown Rice-1 | 194 | 77 | Dantewada | 18.84563 | 81.383933 | 519.14 |
| 63 | Arokhutu | 195 | 78 | Bijapur | 18.79771 | 80.812864 | 327.68 |
| 64 | Brown Rice-2 | 198 | 79 | Dantewada | 18.84563 | 81.383933 | 519.14 |
| 65 | Dubraj | 199 | 80 | Balod | 20.731132 | 81.202306 | 331.00 |
| 66 | HathiPanjra | 201 | 81 | Kondagaon | 19.595851 | 81.663777 | 591.72 |
| 67 | Modipeera | 204 | 82 | Narayanpur | 19.723535 | 81.242411 | 556.68 |
| 68 | Petgadi | 205 | 83 | Dantewada | 18.84563 | 81.383933 | 519.14 |
| 69 | Ramlaxman | 207 | 84 | Dantewada | 18.84563 | 81.383933 | 519.14 |
| 70 | Raja Banga | 208 | 85 | Dantewada | 18.84563 | 81.383933 | 519.14 |
| 71 | Kari Gilash | 209 | 86 | Dhamtari | 20.7015 | 81.554158 | 326.93 |
| 72 | Muni Bhog | 211 | 87 | Bijapur | 18.79771 | 80.812864 | 327.68 |
| 73 | SuaPankhi | 212 | 88 | Dhamtari | 20.7015 | 81.554158 | 326.93 |
| 74 | Mala Gauri | 213 | 89 | Dhamtari | 20.7015 | 81.554158 | 326.93 |
| 75 | DokraDokri | 219 | 90 | Dhamtari | 20.7015 | 81.554158 | 326.93 |
| 76 | NariyalJhoba | 220 | 91 | Dhamtari | 20.7015 | 81.554158 | 326.93 |
| 77 | Sindursal | 225 | 92 | Dantewada | 18.84563 | 81.383933 | 519.14 |
| 78 | Chhindmauri | 226 | 93 | Koria | 23.38755 | 82.388578 | 667.25 |
| 79 | Sugandha | 227 | 94 | Janjgir | 22.0105407 | 82.5726805 | 272.65 |
| 80 | Dandrice | 231 | 95 | Jashpur | 22.78745 | 83.847301 | 495.00 |
| 81 | BansveeraDhan | 239 | 96 | Kanker | 20.272713 | 81.48831 | 400.05 |
| 82 | BeedelaDhan | 246 | 98 | Kanker | 20.272713 | 81.48831 | 400.05 |
| 83 | Sonagathi-2 | 250 | 99 | Balrampur | 23.611174 | 83.610896 | 521.45 |
| 84 | PhalodDhan | 257 | 100 | Narayanpur | 19.723535 | 81.242411 | 556.68 |
| 85 | LalmaDhan | 260 | 101 | Bijapur | 18.79771 | 80.812864 | 327.68 |
| 86 | BaiganiDhan | 265 | 102 | Dantewada | 18.84563 | 81.383933 | 519.14 |
| 87 | MaranDhan | 266 | 103 | Dantewada | 18.84563 | 81.383933 | 519.14 |
| 88 | LoktiMusi | 271 | 104 | Kondagaon | 19.595851 | 81.663777 | 591.72 |
| 89 | AsamChudi | 272 | 105 | Narayanpur | 19.723535 | 81.242411 | 556.68 |
| 90 | Jana Dhan | 280 | 106 | Dantewada | 18.84563 | 81.383933 | 519.14 |
| 91 | RelaDhan | 284 | 107 | Dantewada | 18.84563 | 81.383933 | 519.14 |
| 92 | Jhunuprash | 292 | 108 | Koria | 23.38755 | 82.388578 | 667.25 |
| 93 | OdhaDhanBanarsi | 293 | 109 | Rajnandgaon | 21.0971034 | 81.0302222 | 309.68 |
| 94 | Lochai | 295 | 111 | Jashpur | 22.78745 | 83.847301 | 495.00 |
| 95 | GadurSela | 299 | 112 | Kondagaon | 19.595851 | 81.663777 | 591.72 |
| 96 | KareniDhan | 312 | 113 | Jashpur | 22.78745 | 83.847301 | 495.00 |
| 97 | Govardhan Kali Kamod 2 | 316 | 115 | Mungeli | 22.068542 | 81.685681 | 283.25 |
| 98 | Loindi | 318 | 116 | Koria | 23.38755 | 82.388578 | 667.25 |
| 99 | Santio | 322 | 117 | Dhamtari | 20.7015 | 81.554158 | 326.93 |
| 100 | Parra Dhan | 327 | 118 | Dhamtari | 20.7015 | 81.554158 | 326.93 |
| 101 | Godadani | 328 | 119 | Sitapur | 22.77927 | 83.491024 | 595.41 |
| 102 | Ramshri | 336 | 120 | Dhamtari | 20.7015 | 81.554158 | 326.93 |
| 103 | Danwar | 342 | 121 | Dhamtari | 20.7015 | 81.554158 | 326.93 |
| 104 | ChatiyaNakhi | 344 | 122 | Kondagaon | 19.595851 | 81.663777 | 591.72 |
| 105 | BhathaMasri | 358 | 123 | Kondagaon | 19.595851 | 81.663777 | 591.72 |
| 106 | Kanchan | 361 | 124 | Dhamtari | 20.7015 | 81.554158 | 326.93 |
| 107 | BaigaSeeng | 375 | 125 | Dhamtari | 20.7015 | 81.554158 | 326.93 |
| 108 | MohlaiBanko | 394 | 127 | Dhamtari | 20.7015 | 81.554158 | 326.93 |
| 109 | SutaiDhan | 395 | 128 | Dhamtari | 20.7015 | 81.554158 | 326.93 |
| 110 | Bhujnin | 403 | 130 | Dhamtari | 20.7015 | 81.554158 | 326.93 |
| 111 | Manki | 407 | 131 | Dhamtari | 20.7015 | 81.554158 | 326.93 |
| 112 | Sadachar | 408 | 132 | Dhamtari | 20.7015 | 81.554158 | 326.93 |
| 113 | Mahabaikoni | 418 | 133 | Dhamtari | 20.7015 | 81.554158 | 326.93 |
| 114 | Khajoor | 419 | 134 | Dhamtari | 20.7015 | 81.554158 | 326.93 |
| 115 | Kumhdayin | 420 | 135 | Dhamtari | 20.7015 | 81.554158 | 326.93 |
| 116 | BhejrimaiDhan | 427 | 136 | Dhamtari | 20.7015 | 81.554158 | 326.93 |
| 117 | RatanChudi | 430 | 137 | Kondagaon | 19.595851 | 81.663777 | 591.72 |
| 118 | Panwar | 433 | 138 | Dhamtari | 20.7015 | 81.554158 | 326.93 |
| 119 | Chhindmauri | 435 | 139 | Ambikapur | 23.116444 | 83.196121 | 603.00 |
| 120 | Rani Parewa | 436 | 140 | Kondagaon | 19.595851 | 81.663777 | 591.72 |
| 121 | Mejhri | 442 | 142 | Dhamtari | 20.7015 | 81.554158 | 326.93 |
| 122 | Bodibaja | 444 | 143 | Dhamtari | 20.7015 | 81.554158 | 326.93 |
| 123 | Jela | 447 | 144 | Kondagaon | 19.595851 | 81.663777 | 591.72 |
| 124 | Badshabhog Selection-1 | 448 | 145 | Raipur | 21.2513844 | 81.6296413 | 296.98 |
| 125 | Kari Alcha | 449 | 146 | Dhamtari | 20.7015 | 81.554158 | 326.93 |
| 126 | Anjaniya | 455 | 148 | Dhamtari | 20.7015 | 81.554158 | 326.93 |
| 127 | BaktiChudi | 456 | 149 | Kondagaon | 19.595851 | 81.663777 | 591.72 |
| 128 | JhilliSafri | 458 | 150 | Dhamtari | 20.7015 | 81.554158 | 326.93 |
| 129 | Nanded | 460 | 151 | Dhamtari | 20.7015 | 81.554158 | 326.93 |
| 130 | TuriyaKhudig | 465 | 152 | Dantewada | 18.84563 | 81.383933 | 519.14 |
| 131 | Antarved | 466 | 153 | Dhamtari | 20.7015 | 81.554158 | 326.93 |
| 132 | Rang Chudi | 467 | 154 | Dhamtari | 20.7015 | 81.554158 | 326.93 |
| 133 | MotaChudi | 469 | 155 | Dhamtari | 20.7015 | 81.554158 | 326.93 |
| 134 | B.D. Safri-2 | 471 | 156 | Dhamtari | 20.7015 | 81.554158 | 326.93 |
| 135 | KharikhaKuchi | 472 | 157 | Dhamtari | 20.7015 | 81.554158 | 326.93 |
| 136 | MemriKhedi | 473 | 158 | Dhamtari | 20.7015 | 81.554158 | 326.93 |
| 137 | Ankapalli | 476 | 159 | Dhamtari | 20.7015 | 81.554158 | 326.93 |
| 138 | Samarlengda | 477 | 160 | Dhamtari | 20.7015 | 81.554158 | 326.93 |
| 139 | Mayath | 479 | 161 | Dhamtari | 20.7015 | 81.554158 | 326.93 |
| 140 | MotaSafri | 480 | 162 | Dhamtari | 20.7015 | 81.554158 | 326.93 |
| 141 | Kalinga | 481 | 163 | Bijapur | 18.79771 | 80.812864 | 327.68 |
| 142 | Bhusu | 482 | 164 | Dhamtari | 20.7015 | 81.554158 | 326.93 |
| 143 | Kabeli | 483 | 165 | Dhamtari | 20.7015 | 81.554158 | 326.93 |
| 144 | Gatuvan | 485 | 166 | Dhamtari | 20.7015 | 81.554158 | 326.93 |
| 145 | Baikoni | 488 | 167 | Dhamtari | 20.7015 | 81.554158 | 326.93 |
| 146 | ChinniParas | 490 | 168 | Dhamtari | 20.7015 | 81.554158 | 326.93 |
| 147 | Jalsinga | 491 | 169 | Dhamtari | 20.7015 | 81.554158 | 326.93 |
| 148 | Agni Fag | 493 | 170 | Dhamtari | 20.7015 | 81.554158 | 326.93 |
| 149 | Lalapana | 494 | 171 | Dhamtari | 20.7015 | 81.554158 | 326.93 |
| 150 | BahalBinjo | 495 | 172 | Kondagaon | 19.595851 | 81.663777 | 591.72 |
| 151 | Kari Grass | 496 | 173 | Kondagaon | 19.595851 | 81.663777 | 591.72 |
| 152 | Asam Chudi-2 | 498 | 174 | Bijapur | 18.79771 | 80.812864 | 327.68 |
| 153 | Tulsi Mala | 499 | 175 | Ambikapur | 23.116444 | 83.196121 | 603.00 |
| 154 | Surmatiya | 503 | 176 | Dhamtari | 20.7015 | 81.554158 | 326.93 |
| 155 | Manmohan | 506 | 177 | Dhamtari | 20.7015 | 81.554158 | 326.93 |
| 156 | Jalgundi | 507 | 178 | Dhamtari | 20.7015 | 81.554158 | 326.93 |
| 157 | HathiPinjara | 510 | 179 | Dhamtari | 20.7015 | 81.554158 | 326.93 |
| 158 | ChurlaiBanko | 511 | 180 | Dhamtari | 20.7015 | 81.554158 | 326.93 |
| 159 | Safri-17 | 514 | 181 | Dhamtari | 20.7015 | 81.554158 | 326.93 |
| 160 | Rani Kajar | 515 | 182 | Kondagaon | 19.595851 | 81.663777 | 591.72 |
| 161 | ParwatKal | 518 | 183 | Dhamtari | 20.7015 | 81.554158 | 326.93 |
| 162 | Jhoomar | 519 | 184 | Dhamtari | 20.7015 | 81.554158 | 326.93 |
| 163 | Asam Chudi-3 | 520 | 185 | Kondagaon | 19.595851 | 81.663777 | 591.72 |
| 164 | Jalkeshar | 521 | 186 | Dhamtari | 20.7015 | 81.554158 | 326.93 |
| 165 | Sudama | 524 | 187 | Dhamtari | 20.7015 | 81.554158 | 326.93 |
| 166 | Hajan | 525 | 188 | Dhamtari | 20.7015 | 81.554158 | 326.93 |
| 167 | Ikkopatla | 526 | 189 | Dhamtari | 20.7015 | 81.554158 | 326.93 |
| 168 | HarunaMasri | 528 | 190 | Dhamtari | 20.7015 | 81.554158 | 326.93 |
| 169 | Kosawari | 530 | 191 | Bijapur | 18.79771 | 80.812864 | 327.68 |
| 170 | Bansgathi | 531 | 192 | Dhamtari | 20.7015 | 81.554158 | 326.93 |
| 171 | BhataNakhi | 532 | 193 | Dhamtari | 20.7015 | 81.554158 | 326.93 |
| 172 | Masri | 533 | 194 | Dhamtari | 20.7015 | 81.554158 | 326.93 |
| 173 | Ikkomota | 537 | 195 | Dhamtari | 20.7015 | 81.554158 | 326.93 |
| 174 | Gaurimala | 539 | 196 | Dhamtari | 20.7015 | 81.554158 | 326.93 |
| 175 | Ramjhilli | 549 | 197 | Dhamtari | 20.7015 | 81.554158 | 326.93 |
| 176 | Sichar | 550 | 198 | Dhamtari | 20.7015 | 81.554158 | 326.93 |
| 177 | Rajabangla | 551 | 199 | Dhamtari | 20.7015 | 81.554158 | 326.93 |
| 178 | Majori | 555 | 200 | Dhamtari | 20.7015 | 81.554158 | 326.93 |
| 179 | Hardigathi | 557 | 201 | Kondagaon | 19.595851 | 81.663777 | 591.72 |
| 180 | Ramlaxman | 567 | 202 | Dhamtari | 20.7015 | 81.554158 | 326.93 |
| 181 | Korma | 568 | 203 | Dantewada | 18.84563 | 81.383933 | 519.14 |
| 182 | Dowana | 569 | 204 | Dhamtari | 20.7015 | 81.554158 | 326.93 |
| 183 | Luchai- 2 | 571 | 205 | Raigarh | 21.8974003 | 83.3949632 | 225.99 |
| 184 | Maidubraj | 572 | 206 | Dhamtari | 20.7015 | 81.554158 | 326.93 |
| 185 | Bhaisapuchhi | 574 | 207 | Dhamtari | 20.7015 | 81.554158 | 326.93 |
| 186 | Pancho | 578 | 208 | Dhamtari | 20.7015 | 81.554158 | 326.93 |
| 187 | Bhata Masri-2 | 587 | 209 | Dhamtari | 20.7015 | 81.554158 | 326.93 |
| 188 | Gomti | 593 | 210 | Balrampur | 23.611174 | 83.610896 | 521.45 |
| 189 | Sonkharcha | 594 | 211 | Balrampur | 23.611174 | 83.610896 | 521.45 |
| 190 | Katrani-4 | 599 | 212 | Durg | 21.1904494 | 81.28491689 | 296.7 |
| 191 | Katrani-7 | 602 | 213 | Durg | 21.1904494 | 81.28491689 | 296.7 |
| 192 | KondhaKoya | 611 | 215 | Durg | 21.1904494 | 81.28491689 | 296.7 |

Note: # denotes the locally adapted released cultivar of rice.

Source of coordinates: https://mynasadata.larc.nasa.gov/latitudelongitude-finder/
